# Severe Chronic Traumatic Encephalopathy in a US Naval Special Warfare Combatant Crewman

**DOI:** 10.1001/jamanetworkopen.2025.17686

**Published:** 2025-06-26

**Authors:** David S. Priemer, C. Harker Rhodes, Gregory W. Stewart, Joaquin Villar, Clifton L. Dalgard, Daniel P. Perl

**Affiliations:** 1 Department of Pathology, Uniformed Services University School of Medicine, Bethesda, Maryland; 2 Department of Anatomy, Physiology and Genetics, Uniformed Services University School of Medicine, Bethesda, Maryland; 3 Department of Pediatrics, Uniformed Services University School of Medicine, Bethesda, Maryland; 4 Department of Defense/Uniformed Services University Brain Tissue Repository, US Department of Defense, Bethesda, Maryland; 5 The American Genome Center, Center for Military Precision Health, Uniformed Services University of the Health Sciences, Bethesda, Maryland; 6 The Henry M. Jackson Foundation for Advancement in Military Medicine Inc, Bethesda, Maryland; 7 Tulane University School of Medicine, New Orleans, Louisiana; 8 Tulane University Center for Brain Health, New Orleans, Louisiana

## Abstract

**Importance:**

Chronic traumatic encephalopathy (CTE) is a tauopathy mostly observed following a history of repeated impact-type head injury, usually from contact sports. The overwhelming majority of published, high-stage CTE cases are in older, former high-level contact sport athletes. CTE was infrequent in a large case series of brains donated by military service members, wherein it was most often of minimal severity, while being unanimously present in former contact sport participants.

**Objective:**

To describe the neuropathological examination of a brain donated from a US Naval special warfare combatant crewman (SWCC) who had a 12-year career.

**Design, Setting, and Participants:**

This case study of a whole brain specimen donated after death from a 44.9-year-old military donor with multiple deployments was conducted at a US biorepository dedicated to studies of brains donated from deceased military personnel. The detailed neuropathological examination included comprehensive molecular genetic testing.

**Exposure:**

SWCC are trained to execute high-risk warfare and reconnaissance missions typically operating small, mobile, fast watercraft in numerous maritime environments, including operation in the high seas. In this context, SWCC sustain repetitive physical forces to the body from high-speed boat impacts with waves, which may number in the millions.

**Main Outcomes and Measures:**

Postmortem diagnosis for CTE using the National Institute of Neurological Disorders and Stroke–National Institute of Biomedical Imaging and Bioengineering (NINDS-NIBIB) consensus criteria as well as the McKee staging system; molecular genetic assessment for neurodegenerative disease.

**Results:**

The case specimen was from a male 44.9-year-old who served a 12.2-year SWCC career from ages 28.9 to 41.1 years. Following his last deployment, he experienced posttraumatic stress disorder, alcohol misuse, migraines, and impaired cognition. He died by suicide. Neuropathological evaluation revealed severe CTE (high CTE according to NINDS-NIBIB consensus criteria; McKee Stage IV of IV), with extensive pathology involving numerous brain regions. Molecular genetic testing for neurodegenerative disease risk was negative.

**Conclusions and Relevance:**

In this neuropathological case study of a 44-year-old former SWCC, we found severe CTE. Given the severity of the pathology at a relatively young age, we consider that exposures of a SWCC career, including repetitive physical forces applied to the head from high-speed boat impacts with waves, may be sufficient to promote or otherwise contribute to CTE development.

## Key Points

**Question:**

Can exposures to dynamic forces sustained by US Naval special warfare combatant crewmen (SWCC), including repetitive, high-speed boat impacts with waves, predispose them to chronic traumatic encephalopathy (CTE)?

**Findings:**

This case study neuropathological evaluation of the brain from a deceased, 44.9-year-old SWCC with a 12-year career revealed high-stage CTE. A 204-gene panel assessing for neurodegenerative disease risk was negative.

**Meaning:**

Although the overwhelming majority of published, high-stage CTE cases involve older adults who were formerly contact sport athletes, the extent of pathology encountered in this case suggests the possibility that exposures within a SWCC career may have been sufficient to promote severe CTE.

## Teaser

This case study of a whole brain specimen from a deceased US military donor exposed to repetitive high-speed boat impacts with waves presents the results of a neuropathological evaluation for chronic traumatic encephalopathy.

## Introduction

Chronic traumatic encephalopathy (CTE) is an acquired tauopathy most characteristically associated with repetitive impact-type traumatic brain injury, particularly in the context of contact sports participation (eg, American football, boxing).^1,2^ In 2022, our laboratory, the Department of Defense/Uniformed Services University Brain Tissue Repository (DoD-USU BTR), reported results of the comprehensive neuropathological examination of 225 consecutively donated brains from deceased active duty and former military personnel for the neuropathology of CTE.^3^ The series represented a diversity of service members with broad ranges of traumatic brain injury exposures (both in civilian and military contexts) and a broad range of clinical symptomatology (ie, psychiatric disease, alcohol and/or substance abuse, and suicidality). In summary, we reported 10 cases of CTE neuropathology within this series (4.4%), most of which were mild or minimally diagnostic in nature, raising the question of clinical significance. All 10 cases of CTE were associated with prior participation in contact sports, such that 10 of the 60 former contact sports athletes in the series had CTE neuropathology, while zero of the 165 nonathletes had CTE neuropathology. Military TBI exposures, and particularly blast exposures, had the lowest numerical relationship with CTE neuropathology. Furthermore, and although our study was insufficiently powered to evaluate the association of CTE with factors such as psychiatric disease, alcohol and/or substance abuse, and suicide, pathologic features of CTE were not found in the large majority of cases with these factors. In conclusion, we reported that CTE neuropathology was infrequent in a large series of military service members, was more common to a history of impact-type TBI from contact sports involvement as opposed to military service and blast exposure, was not identified in the large majority of service members exhibiting neuropsychiatric features, and was typically minimal-to-mild in nature and thus likely not of clinical significance. In the 3 years that have passed since the publication of this case series, the experience of the DoD-USU BTR with respect to the frequency and severity of CTE in continued brain donations has largely remained unchanged.

US special warfare combatant crewman (SWCC) personnel make up a relatively small and exclusive group of elite military service members within the Special Operations Forces. SWCC personnel operate small, specialized boats (examples and/or alternative nomenclature: high-speed boats, fast boats, fast attack or assault craft, fast patrol boats, expeditionary fast transport craft) at high velocities (50 knots or more, or 93 kilometers/h or more), and sustain numerous, repetitive, so-called “shock” forces as the boats repeatedly impact with waves during operation.^4,5,6,7^ Impacts can frequently occur with waves up to 8 feet (2.44 m) in height, and separate studies and inquiries show that wave impacts often produce between 2 and 15 g-forces, but many are also much greater (20 g-forces or more).^4,5,6,7^ Clinically, this occupational exposure has been associated with high rates of major musculoskeletal injuries, such as large fractures, and a hospitalization rate that is 5 times the average rate of active duty Navy personnel.^8^ Neuropsychiatric sequalae have also been implicated.^8,9,10,11^ Herein we describe the evaluation of the brain from a symptomatic SWCC whose profound CTE neuropathology represents a substantial outlier within the experience of the DoD-USU BTR, and may raise concern for exposures related to this particular military occupation and risk of CTE development.

## Methods

### Case History

This case history was acquired by available medical records, military records, online public records, and by retrospective, semi-structured interviews with the next of kin (immediate family). The decedent was a 44.9-year-old man and veteran of the Navy. Over the course of a 12.2-year Naval career from ages 28.9 to 41.1 years, the decedent served in the Special Forces as a SWCC. He served at least 4 combat deployments as a gunman, driver, and captain. In his position, he worked with many large-caliber weapons and had a known history of blast exposure. Following his last deployment, he was diagnosed with severe posttraumatic stress disorder, and drank alcohol heavily. For 10 years prior to death, the decedent also experienced severe migraine headaches and chronic sleep impairment. In at least the 6 months prior to death, he was described as not thinking well, with difficulty putting sentences together when writing, and had a rapid mental decline associated with symptoms of hallucinations, paranoia, and suicidal ideations. The decedent died by suicide via a self-inflicted gunshot wound to the chest.

Of note, prior to joining the military, the decedent was a baseball player whose career had extended to 4 years in the Minor Leagues (ages 20 to 23 years) and 2 years in independent leagues (ages 24 to 25 years); he primarily played as a catcher. Our records do not indicate a history of TBI sustained during his years of baseball participation, and a history of head injury related to baseball was denied by the next-of-kin. In addition, our records similarly do not indicate TBIs sustained from civilian activities unrelated to contact sports, such as those that may occur from motor vehicle accidents or falls, and history of these was similarly denied by the next-of-kin.

### Neuropathological Examination

Following death, the decedent’s whole brain was donated by next-of-kin to the DoD-USU BTR. The interval from the official date and time of death to brain retrieval and fixation in formalin (postmortem interval) was 69.5 hours. The decedent had been in refrigeration since the day of death, and postmortem examination took place on the third day following; as such, the majority of the postmortem interval occurred under refrigeration. The brain was received for examination by the DoD-USU BTR following 2 weeks of formalin fixation. Following gross examination, samples taken for histologic examination included: orbitofrontal regions (bilateral), dorsolateral prefrontal regions (bilateral), superior and middle temporal gyri, parietal lobe (bilateral), occipital lobe (visual cortex), anterior cingulate gyrus and corpus callosum, insula, basal ganglia, thalamus and hypothalamus (multiple samples to include mamillary body and subthalamic nucleus), amygdala and adjacent mesial temporal cortex, hippocampi with adjacent mesial temporal cortex (bilateral), midbrain, pons, medulla, and cerebellum.

Each formalin-fixed tissue sample was processed into paraffin blocks and 5-μm thick sections were prepared for histological evaluation. Hematoxylin and eosin staining was performed on sections from each sample. Sections for each sample were also stained for phosphorylated tau (ptau; AT8, mouse anti-human monoclonal antibody, Thermo Scientific), glial fibrillary acidic protein (GA5 antibody, Leica Biosystems), Aβ protein (anti-β-amyloid 1-16 antibody, clone 6E10, BioLegend), and amyloid precursor protein (APP; anti-APP A4 antibody, a.a. 66 to 81 of APP [NT], clone 22C11, Milipore-SIGMA). Sections from select regions were immunostained stained for α-synuclein (recombinant anti-α synuclein [Phosphor S129] antibody [EP1536Y], Abcam) and transactive response DNA binding protein 43 (TDP-43; recombinant anti–TDP-43 antibody [EPR 5810], Abcam).

### Molecular Genetic Testing: Targeted Panel Sequence Profiling

Total nucleic acids were isolated from brain tissue (cerebellum, following formalin fixation) using the total nucleic acid protocol (Covaris, LLC) with minor modifications for automation. Genomic DNA was used as input for library preparation and capture using a DNA preparation kit with enrichment and a sequencing panel (Illumina, Inc). Sequencing libraries were assessed for quality before sequencing (Illumina, Inc). Raw sequencing data were aligned, variant called and annotated using Dragen version 4.2.4 (Illumina, Inc). Variant interpretation was conducted by a clinical molecular geneticist with variant prioritization and classification using a genomizer with manual confirmation.

### Ethical Approval Statement

All brains at the DoD-USU BTR are donated for neuropathological examination and use in research and scientific publication by next-of-kin or appropriate legal representatives by written consent, which in this case also included consent for genetic testing. This study was conducted under a protocol that has been approved by the USU institutional review board.

## Results

### Neuropathological Examination

The weight of the fixed brain was 1489 g (mean [SD] weight for age and sex, 1430 [20] g).^12^ Gross examination was that of a well-developed adult brain, and was notable only for a cavum septum pellucidum and slight pallor of the substantia nigra and locus coeruleus. Gross examination demonstrated good tissue integrity and was without notable or otherwise characteristic postmortem, decompositional changes.

**Figure 1.**
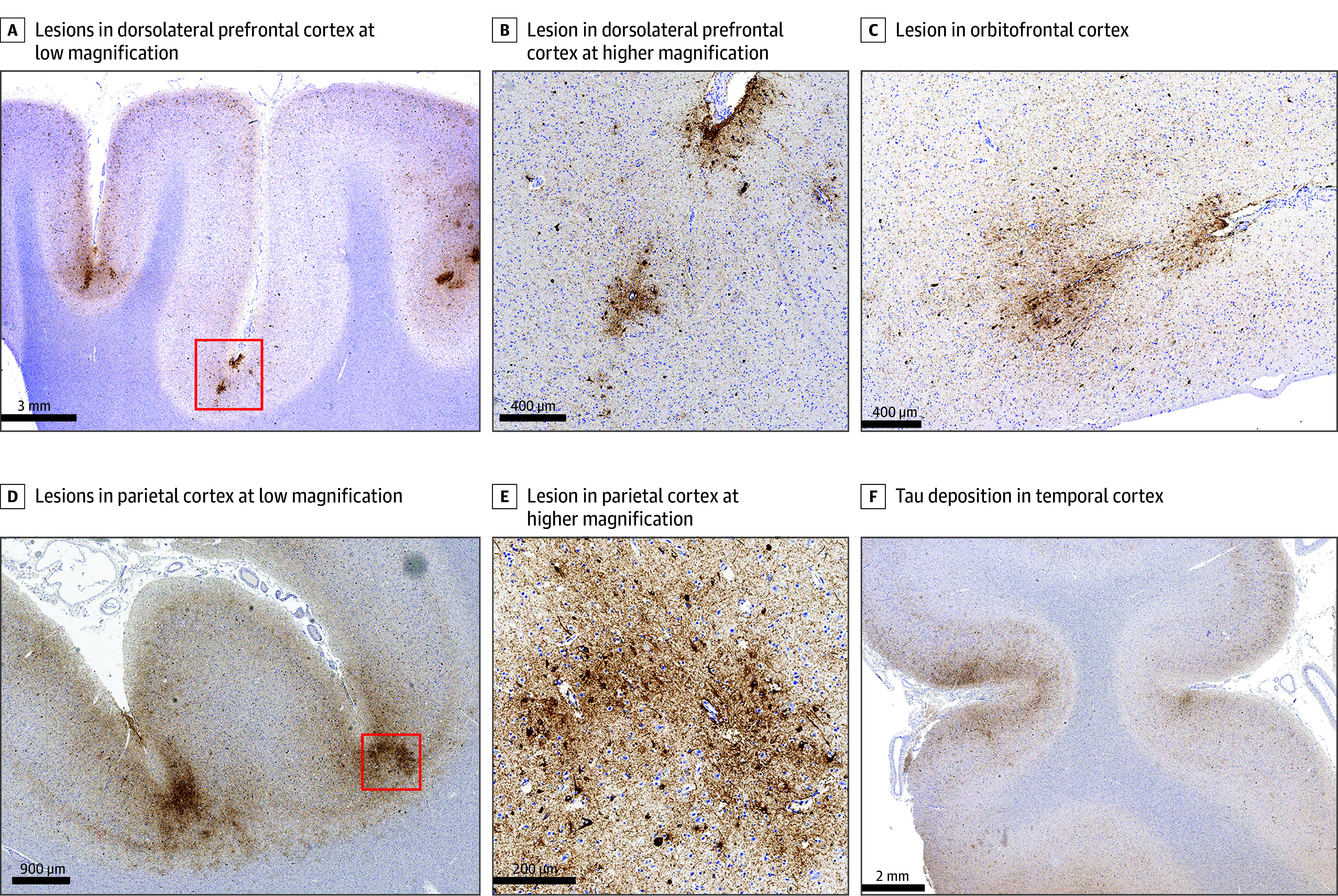
Severe, Multilobar Chronic Traumatic Encephalopathy (CTE) in a Naval Servicemember, AT8 Immunohistochemistry for Phosphorylated Tau Protein A, Demonstrates sulcal depth tau lesions characteristic of CTE in contiguous sulci at low magnification (scale bar = 3 mm) in a section from the dorsolateral prefrontal cortex; red box corresponds to region magnified in B. B, Demonstrates a pathognomonic lesion for CTE from the dorsolateral prefrontal cortex (corresponding to the region of the red box in A) at higher magnification (scale bar = 400 μm), characterized particularly by perivascular neuronal tau accumulation at a cortical sulcal depth. C, Additional example of another CTE lesion from a sample of the orbitofrontal cortex (scale bar = 400 μm). D, Demonstrates CTE lesions in contiguous sulci in a sample from the parietal lobe at low magnification (scale bar = 900 μm), red box corresponds to the region magnified in E. E, Demonstrates an extensive perivascular neuronal tau accumulation at a sulcal depth in the parietal cortex (corresponding to the region of the red box in D) at higher magnification (scale bar = 200 μm). F, Demonstrates extensive generalized tau deposition in the temporal cortex, with some concentration at the sulcal depths, at low magnification (scale bar = 2 mm).

**Figure 2.**
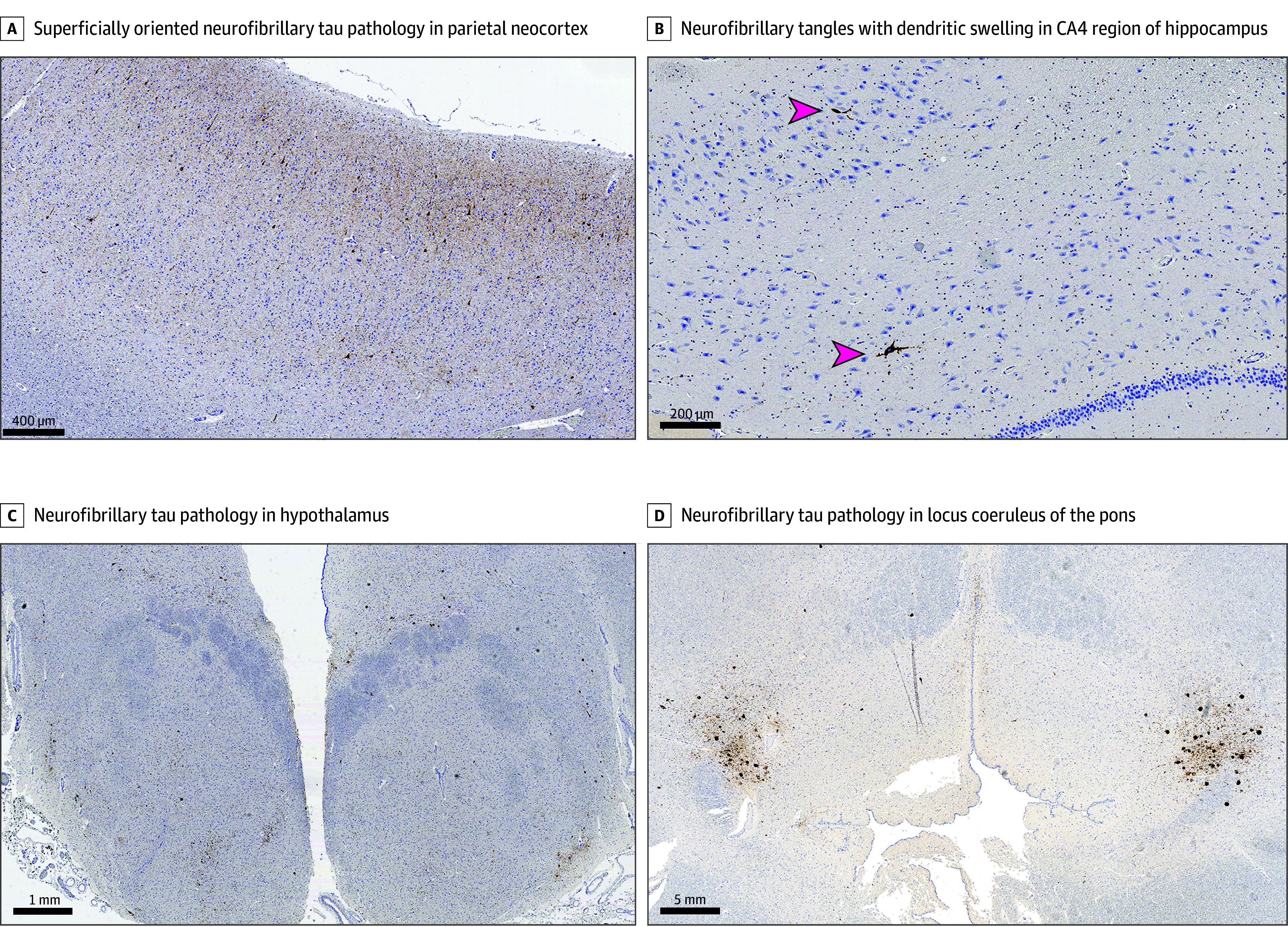
Extensive Tau Pathology Corresponding to Secondary Characteristics of Chronic Traumatic Encephalopathy (CTE), AT8 Immunohistochemistry for Phosphorylated Tau Protein A, Beyond sulcal depth lesions which are pathognomonic for CTE, this case additionally demonstrated diffuse cortical neuronal tau deposition, particularly in superficial layers of the cortex, which was prominent in the frontal, temporal, and parietal cortices and more minimal in the occipital sampling. This pattern of superficial cortical tau spread is commonly observed in severe cases of CTE; photomicrograph of a section from the parietal neocortex (scale bar = 400 μm). B, Demonstrates prominent neurofibrillary tangles (arrows) with dendritic swellings in the CA4 region of the hippocampus (scale bar = 200 μm), known to be a common secondary characteristic of CTE. C, Demonstrates extensive neurofibrillary tau pathology in the hypothalamus, including the mammillary bodies, at low magnification (scale bar = 1 mm). D, Demonstrates extensive neurofibrillary tau pathology in the locus coeruleus of the pons, at low magnification (scale bar = 5 mm).

Examination of hematoxylin and eosin–stained slides did not reveal evidence of significant autolysis or other postmortem tissue degradation. Examination of AT8-immunostained sections for ptau revealed an extensive tauopathy, with abundant neuronal deposition as neurofibrillary tangles, pretangles, and numerous neuronal threads most severally involving the temporal and parietal neocortex, but also the frontal cortex and, to a minimal degree, the occipital cortex. The cortical involvement was principally characterized by predominant aggregation of ptau in neurons in perivascular distributions at numerous sulcal depths, which is diagnostic of CTE neuropathology (Figure 1).^13^ Several secondary patterns of pathology often associated with CTE were also observed. Namely, numerous neurofibrillary tangles were also noted away from sulcal depths, and particularly in superficial cortical layers. Notable neurofibrillary pathology was also observed in the amygdala, hippocampi (with prominent involvement of the CA4 region), basal forebrain, thalamus, and hypothalamus; in the brainstem the locus coeruleus was severely affected by neurofibrillary pathology, as were the dorsal raphe, and to a lesser degree the substantia nigra and periaqueductal gray matter were also involved (Figure 2). Astroglial ptau was sparingly identified in the cerebral cortex, as well in periventricular sites, particularly the around the temporal horn of the lateral ventricles. With this severe extent of ptau pathology, this case meets criteria as an example of high CTE according to the most recent National Institute of Neurological Disorders and Stroke-National Institute of Biomedical Imaging and Bioengineering consensus (NINDS-NIBIB) criteria for CTE, which is expected to correspond to the most severe stages of disease according to the McKee CTE staging scheme (stages III and IV).^2,13^ According to the McKee staging scheme, this case is classified as stage IV of IV.

**Figure 3.**
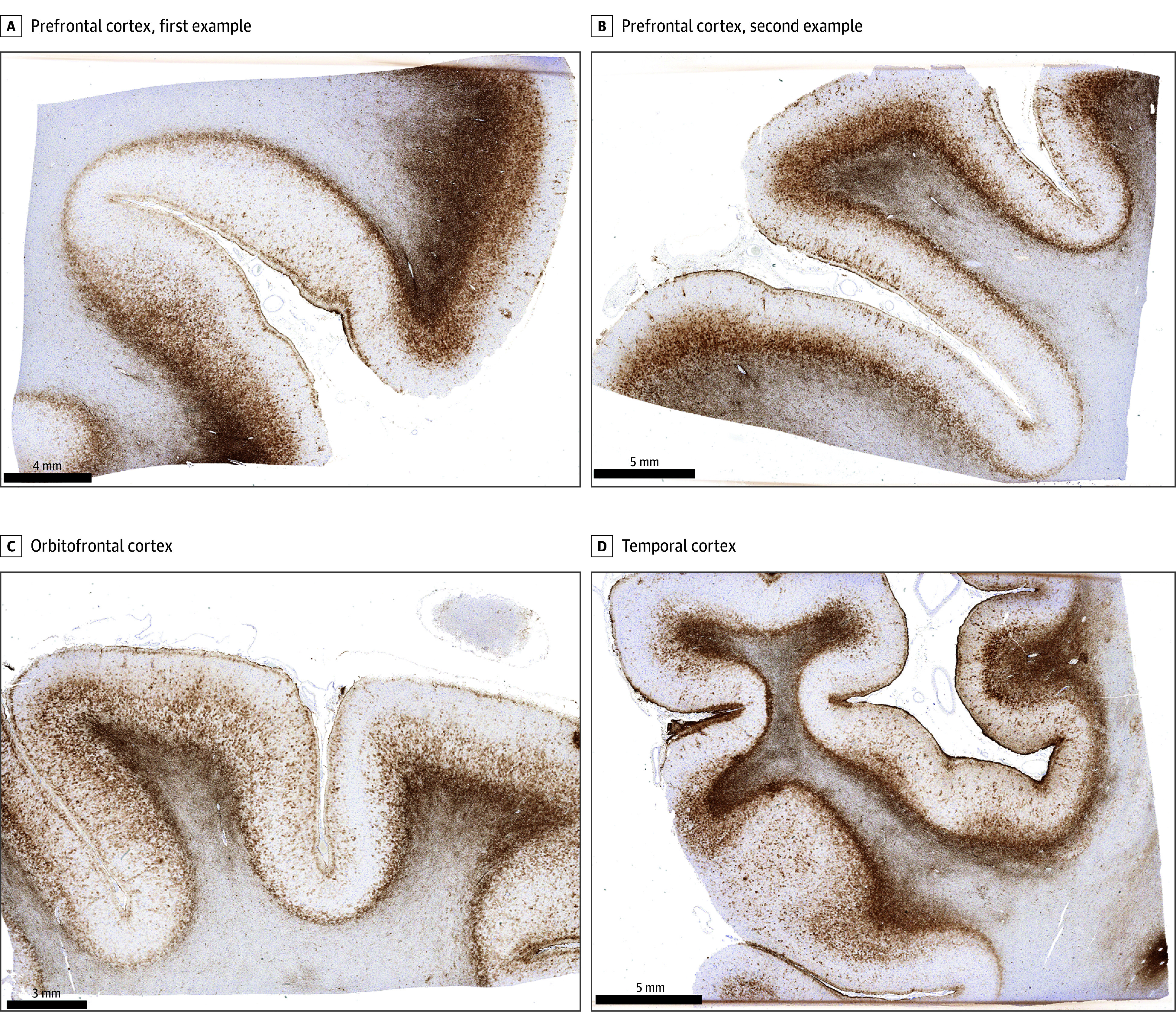
Microscopic Features of Interface Astroglial Scarring A-D (scale bars = 4 mm, 5 mm, 3 mm, and 5 mm, respectively), glial fibrillary acidic protein immunohistochemistry demonstrating substantially increased labeling, corresponding to astroglial scarring, particularly at subpial and gray-white interfaces, in select sections of the dorsolateral prefrontal cortex (A, B), orbitofrontal cortex (C), and temporal cortex (D). This pathology has been most predominantly described in the setting of prior blast-type traumatic brain injury.

Immunohistochemical stains performed on sections from all the aforementioned tissue samples for glial fibrillary acidic protein revealed markedly increased labeling at brain interfaces, particularly in the neocortical gray-white matter junction and subpial regions, compatible with a neuropathological diagnosis of interface astroglial scarring, which our laboratory and collaborators have associated with blast exposure (Figure 3).^14,15^ Immunohistochemistry for APP was negative for evidence of axonal injury. Immunohistochemistry for Aβ protein was negative for evidence of plaque formation or vascular deposition. Immunohistochemistry for α-synuclein performed on sections from select regions (pons, midbrain, cingulate gyrus, amygdala, frontal lobe, temporal lobe) was negative for evidence of Lewy body formation or glial cytoplasmic inclusions. Immunohistochemistry for TDP-43 was negative for pathologic neuronal inclusions or neurites on sections from select regions (amygdala, hippocampus, frontal lobe, temporal lobe).

### Molecular Genetic Testing

Targeted genetic analysis for this case by next generation sequencing resulted in no observable variants of pathogenic classification (ie, likely pathogenic or pathogenic) for 204 genes associated with neurodegenerative disease risk. For example, there were no pathogenic or likely pathogenic variants in the *MAPT* gene to indicate a genetic tauopathy, in genes associated with familial or early onset Alzheimer disease (eg, *APP*, *PSEN1*, *PSEN2*), in *TBK1* as a potential promotor of tau hyperphosphorylation in multiple tauopathies, or in *TMEM106B,* for which the single nucleotide polymorphism rs3173615 has been implicated as a potentially modifying factor in the extent of tauopathy in cases of CTE.^16,17,18,19,20^ A table of all genes tested and associated results can be reviewed in eTable 1 and eTable 2 in Supplement 1. Additionally, *APOE* was genotyped for e3/e3 alleles, corresponding to normal late-onset Alzheimer disease risk.

## Discussion

In the setting of exposure to forces from repetitive high-speed boat impacts with waves, SWCC personnel are exposed to extensive, repeated, whiplash-like jostling of their heads wherein there is often no external head impact, but the brain is distorted from these forces and nonetheless impacts the inside of the skull in the context of rapid acceleration and deceleration dynamics. In addition, and given the high rates of significant musculoskeletal injuries that occur in this military occupational specialty,^8^ the opportunity for multiple true head impacts within the boats or with external objects (eg, jumping fish, branches or other shoreline objects) also cannot be excluded. Reported neuropsychiatric sequalae that may be experienced by SWCC personnel include headaches, nausea, sleepiness or fatigue, loss of visual acuity and hand-eye coordination, headaches, and general “performance degradation.”^8,9,10^ Furthermore, a more recent study by Ullman et al^11^ published in 2022 reported results of an online survey of SWCC personnel in which 72 of 214 (34%) respondents reported a total of 148 episodes of loss of consciousness, and 105 of 214 (49%) reported “events of cognitive impairment because of impact exposure.”

We report a case of severe CTE in a SWCC operator who had a 12-year-long career operating combatant crafts in several capacities. Given that this is a single case, we can make no definitive statement regarding the contribution of the CTE pathology to any clinical symptomatology, particularly given coexistence of other pathology (interface astroglial scarring). However, in comparison with previously published cases concerning military personnel from the DoD-USU BTR,^3^ this case represents a notable exception to our experience with regard to the marked severity of CTE-related lesions identified upon pathological examination and the relatively young age (44 years) of the individual. It should also be noted that the overwhelming majority of individuals whose brains have been characterized in the published medical research with McKee stage IV CTE, or high CTE according to NINDS-NIBIB consensus criteria, are individuals who died at ages older than 60 years (mean age in the 70s), and these cases are exceedingly observed in former elite contact sports athletes, namely American football players or fighters in combative sports such as boxing.^2,21,22,23^

A 1994 report by Roesch et al^4^ provides the most available published insights regarding the extent and magnitude of wave impact events that occur with use of Naval Special Warfare high-speed boats. Within this report, the authors detail studies on the operation of the boats that took place over a 6-day period and 36 hours of high-speed boat operation. The authors documented 8610 boat impacts with waves (239.2 impacts per hour), with the force of impact producing g-forces that ranged from 2 to 25, with 4496 of them (124.9 impacts per hour) associated with g-force measurements of greater than 5. A modest estimate of time spent in boating operation for a SWCC is 1500 to 1800 hours per deployment cycle; however, lengthier cycles may result in 3000 hours or more (G.W.S., phone, April 17, 2025). The decedent in this case, having served at least 4 deployments, would therefore be estimated to have been exposed to boat impacts with waves, associated with both with g-forces greater than 2 and greater than 5, that number in the many hundreds of thousands to millions. We therefore speculate that this volume of sustained boat impacts with waves, and any associated TBI (with or without true impact events), predisposed to CTE development or was otherwise a major contributing factor.

### Limitations

We acknowledge that this is a single case, that other factors could be involved, and therefore that we cannot make definitive conclusions pending examination of many additional SWCC cases. We have therefore taken special precaution with regard to other potential contributory factors toward CTE development. We recognize that an isolated case of CTE in a professional baseball player has been reported in the peer-reviewed literature, and thus that a potential contribution from this activity to CTE in the case we report here cannot be excluded.^21^ However, the severity of disease identified within the only reported case of CTE in a baseball player was mild (McKee stage II, or low CTE according to the NINDS-NIBIB consensus criteria), much unlike the disease identified in the case presented here. Furthermore, lay media articles indicate that the “all-out” playing style of the professional baseball player whose brain was found to have mild CTE was uniquely hazardous in comparison with that of his peers, and notably that this player was “plagued” by numerous head injuries and concussions that ultimately ended his career.^24,25,26^ Such an extensive TBI history relating to baseball is absent in the case we report here. We have also not identified any history of notable TBI events occurring in a civilian context outside of sports, ie, our extensive case review did not reveal a history of TBI from events such as motor vehicle accidents or falls. We also considered other aspects of the decedent’s military career, less specific to a SWCC occupation, such as those involving him having been a machine gunner, and having been exposed to blast. These factors were largely represented in the large, published series of brains from a broad range of military decedents in our repository, and did not appear to be associated with a high rate of CTE, and certainly not with severe CTE.^3^ Importantly, approximately 10% of the cases studied in our published series involved other Special Operations Forces personnel (eg, Army Rangers, Navy SEALs), most of whom served extensively in combat and training roles. Finally, although there is no established genetic risk factor for CTE development, we performed an exhaustive molecular panel for genes to known to be associated with tauopathy and more broadly with neurodegenerative risk, which failed to identify pathogenic variants. We therefore were not able to identify evidence of genetic susceptibility to the presence or extent of tauopathy in this case.

## Conclusions

In this neuropathological case study of a deceased, 44.9-year-old SWCC operator who served a 12-year career with at least 4 combat deployments, we report severe CTE. Given the extent of the CTE pathology identified in this case, and particularly compared with other published data on CTE in military personnel and in the absence of an identifiable genetic risk factor for neurodegenerative disease upon comprehensive molecular testing, it is our interpretation that occupational exposures of a SWCC career, including prolonged and extensive exposure to repetitive physical forces applied to the head from high-speed boat impacts with waves, may be sufficient to promote or otherwise contribute to the development of CTE. While we cannot draw definitive conclusions from a single case, we raise the concern that SWCC personnel, in general, may be exposed to unique physical hazards that can lead to persistent impairment in brain health, including the potential development of severe CTE. Further studies of SWCC personnel are warranted.

## Notes

Supplement 1. **eTable 1.** Gene List: Targeted Genetic Analysis for Dementia Risk **eTable 2.** Gene Variants Identified

Supplement 2.
Data Sharing Statement

## References

[1] McKee AC, Cantu RC, Nowinski CJ, . Chronic traumatic encephalopathy in athletes: progressive tauopathy after repetitive head injury. J Neuropathol Exp Neurol. 2009;68(7):709-735. doi:10.1097/NEN.0b013e3181a9d503 19535999 PMC2945234

[2] McKee AC, Stern RA, Nowinski CJ, . The spectrum of disease in chronic traumatic encephalopathy. Brain. 2013;136(Pt 1):43-64. doi:10.1093/brain/aws307 23208308 PMC3624697

[3] Priemer DS, Iacono D, Rhodes CH, Olsen CH, Perl DP. Chronic traumatic encephalopathy in the brains of military personnel. N Engl J Med. 2022;386(23):2169-2177. doi:10.1056/NEJMoa2203199 35675177

[4] Roesch JR, Curley M, Hart B. Sudden impacts in Naval Special Warfare high-speed boats. Naval Surface Warfare Center report No. CSS/TR-94/38. September 1994. Accessed November 13, 2024. https://combatantcraftcrewman.org/wp-content/uploads/2021/12/Impact-Reports-1.pdf

[5] Kearns SD. Analysis and mitigation of mechanical shock effects on high speed planing boats. Massachusetts Institute of Technology graduate thesis. September 2001. Accessed November 13, 2024. https://dspace.mit.edu/handle/1721.1/8235

[6] Garme K, Burstrom L, Kuttenkeuler J. Measures of vibration exposure for a high-speed craft crew. J Engineering for the Maritime Environment. 2011;225:338-349. doi:10.1177/1475090211418747

[7] Myers SD, Dobbins TD, King S, . Effectiveness of suspension seats in maintaining performance following military high-speed boat transits. Hum Factors. 2012;54(2):264-276. doi:10.1177/0018720811436201 22624292

[8] Ensign W, Hodgdon JA, Prusaczyk WK, Shapiro D, Lipton M. A survey of self-reported injuries among special boat operators. Naval Health Research Center: Technical Report No. 00-48. December 1999. Accessed November 13, 2024. https://apps.dtic.mil/sti/pdfs/ADA421234.pdf

[9] Gollwitzer RM, Peterson RS. Repeated water entry shocks on high-speed planing boats. Naval Surface Warfare Center report No. CSS/TR-96/27. September 1995. Accessed November 13, 2024. https://apps.dtic.mil/sti/tr/pdf/ADA317132.pdf

[10] Griffith MJ. Human Vibration Handbook. Academic Press; 1990.

[11] Ullman J, Hengst D, Carpenter R, Robinson Y. Does military high-speed boat slamming cause severe injuries and disability? Clin Orthop Relat Res. 2022;480(11):2163-2173. doi:10.1097/CORR.0000000000002420 36190503 PMC9556009

[12] Dekaban AS. Changes in brain weights during the span of human life: relation of brain weights to body heights and body weights. Ann Neurol. 1978;4(4):345-356. doi:10.1002/ana.410040410 727739

[13] Bieniek KF, Cairns NJ, Crary JF, ; TBI/CTE Research Group. The Second NINDS/NIBIB Consensus Meeting to Define Neuropathological Criteria for the Diagnosis of Chronic Traumatic Encephalopathy. J Neuropathol Exp Neurol. 2021;80(3):210-219. doi:10.1093/jnen/nlab001 33611507 PMC7899277

[14] Shively SB, Horkayne-Szakaly I, Jones RV, Kelly JP, Armstrong RC, Perl DP. Characterisation of interface astroglial scarring in the human brain after blast exposure: a post-mortem case series. Lancet Neurol. 2016;15(9):944-953. doi:10.1016/S1474-4422(16)30057-6 27291520

[15] Schwerin SC, Chatterjee M, Hutchinson EB, . Expression of GFAP and tau following blast exposure in the cerebral cortex of ferrets. J Neuropathol Exp Neurol. 2021;80(2):112-128. doi:10.1093/jnen/nlaa157 33421075 PMC8453607

[16] Strang KH, Golde TE, Giasson BI. MAPT mutations, tauopathy, and mechanisms of neurodegeneration. Lab Invest. 2019;99(7):912-928. doi:10.1038/s41374-019-0197-x 30742061 PMC7289372

[17] Abreha MH, Ojelade S, Dammer EB, . TBK1 interacts with tau and enhances neurodegeneration in tauopathy. J Biol Chem. 2021;296:100760. doi:10.1016/j.jbc.2021.100760 33965374 PMC8191334

[18] Tanzi RE. The genetics of Alzheimer disease. Cold Spring Harb Perspect Med. 2012;2(10):a006296. doi:10.1101/cshperspect.a006296 23028126 PMC3475404

[19] Cherry JD, Mez J, Crary JF, . Variation in TMEM106B in chronic traumatic encephalopathy. Acta Neuropathol Commun. 2018;6(1):115. doi:10.1186/s40478-018-0619-9 30390709 PMC6215686

[20] Bieniek KF, Ross OA, Cormier KA, . Chronic traumatic encephalopathy pathology in a neurodegenerative disorders brain bank. Acta Neuropathol. 2015;130(6):877-889. doi:10.1007/s00401-015-1502-4 26518018 PMC4655127

[21] McKee AC, Daneshvar DH, Alvarez VE, Stein TD. The neuropathology of sport. Acta Neuropathol. 2014;127(1):29-51. doi:10.1007/s00401-013-1230-6 24366527 PMC4255282

[22] Mez J, Daneshvar DH, Kiernan PT, . Clinicopathological evaluation of chronic traumatic encephalopathy in players of American football. JAMA. 2017;318(4):360-370. doi:10.1001/jama.2017.8334 28742910 PMC5807097

[23] Alexander A, Alvarez VE, Huber BR, . Cortical-sparing chronic traumatic encephalopathy (CSCTE): a distinct subtype of CTE. Acta Neuropathol. 2024;147(1):45. doi:10.1007/s00401-024-02690-5 38407651 PMC11348287

[24] ABC News Network. Ryan Freel had CTE, parents say. December 15, 2013. https://abcnews.go.com/Sports/ryan-freel-cte-parents/story?id=21227773

[25] Barney J. Ryan Freel: Full speed, crashing end. *The Florida Times-Union*. January 19, 2013. Accessed November 13, 2024. https://www.jacksonville.com/story/sports/high-school/2013/01/20/ryan-freel-full-speed-crashing-end/15840808007/

[26] The Associated Press. Ryan Freel, concussion-plagued baseball player, dies at 36. *The New York Times*. December 24, 2012. Accessed November 13, 2024. https://www.nytimes.com/2012/12/25/sports/baseball/ryan-freel-concussion-plagued-baseball-player-dies-at-36.html

