# Supplementary material for: Severe Chronic Traumatic Encephalopathy in a US Naval Special Warfare Combatant Crewman
Source: JAMA Netw Open. 2025 Jun 26;8(6):e2517686. doi: 10.1001/jamanetworkopen.2025.17686 (PMC12203286; doi:10.1001/jamanetworkopen.2025.17686)
Supplement: Supplement 1. — eTable 1. Gene List: Targeted Genetic Analysis for Dementia Risk eTable 2. Gene Variants Identified [file jamanetwopen-e2517686-s001.pdf]

Supplemental Online Content

Priemer DS, Rhodes CH, Stewart GW, Villar J, Dalgard CL, Perl DP. Severe chronic traumatic encephalopathy in a United States naval special warfare combatant crewman. *JAMA Netw Open.* 2025;8(6):e2517686. doi:10.1001/jamanetworkopen.2025.17686

eTable 1. Gene List: Targeted Genetic Analysis for Dementia Risk

eTable 2. Gene Variants Identified

This supplemental material has been provided by the authors to give readers additional information about their work.

© 2025 Priemer DS et al. *JAMA Network Open*.

eTable 1. Gene List: Targeted Genetic Analysis for Dementia Risk

ABCA7	BIN1	DDHD1	HEXA	MS4A6E	PRKN	SND1	TOMM40
ABI3	BLMH	DNAJC13	HNRNPA1	NEFH	PRKRA	SOD1	TOR1A
ACMSD	BTNL2	DNAJC6	HNRNPA2B1	NEK1	PRNP	SORL1	TP53INP1
ADAM10	C9orf72	DNMT1	HNRNPD	NIPA1	PRPH	SPAST	TREM2
ADORA1	CASS4	DPYSL3	HSPA9	NLRC5	PRRT2	SPG11	TREML2
AK9	CD2AP	EIF4G1	HTRA2	NME8	PSEN1	SPR	TRIP4
AKT1	CD33	ELAVL1	INPP5D	NOTCH3	PSEN2	SQSTM1	TRPM2
ALAD	CELF1	EP300	ITM2B	OPTN	PSMB7	SS18L1	TRRAP
ALS2	CHCHD10	EPHA1	KCNV2	P4HB	PTEN	STK36	TUBA4A
ANAPC7	CHCHD2	EPHA4	KIF5A	PANK2	PTK2B	SV2A	TYROBP
ANG	CHGB	ERBB4	LAMA1	PARK7	RAB29	SYNJ1	UBQLN2
ANKRD13A	CHMP2B	ERLIN1	LCT	PDE8B	RAB38	TAF1	UCHL1
ANO3	CHRM1	EWSR1	LIMD1	PDIA3	RAB39B	TAF15	UNC13A
ANXA11	CHRNA4	FBXO7	LMNB1	PFN1	RIN3	TARDBP	UNC5C
APEX1	CLCN6	FERMT2	LRRK1	PICALM	RINL	TBK1	VAPB
APOE	CLU	FIG4	LRRK2	PINK1	RUNDC3A	TBP	VCP
APP	COL12A1	FLNC	LUM	PLA2G4C	SARM1	TF	VEGFA
APTX	COL19A1	FOXN3	MAPT	PLA2G6	SCARB2	TFG	VPS13C
ATM	CR1	FUS	MARK2	PLCG2	SETX	TH	VPS35
ATP13A2	CRYM	GAK	MARK4	PLD3	SGTA	THAP1	VPS53
ATP1A3	CSF1R	GBA	MATR3	PMEL	SIGMAR1	THSD7B	XPR1
ATP6AP2	CTNNA3	GCH1	MEF2C	PODXL	SLC24A4	TIA1	ZCWPW1
ATP7B	CTSC	GIGYF2	METTL22	POLG	SLC5A9	TMEM106B	ZNF512B
ATP8B3	CTSD	GLE1	MGA	PON1	SLC6A3	TMEM230	
ATXN2	DAO	GRN	MS4A4A	PON2	SNCA	TNK2	
BDNF	DCTN1	GTF2H4		PON3	SNCAIP	TNR	

© 2025 Priemer DS et al. *JAMA Network Open*.

eTable 2. Gene Variants Identified

Gene	Transcript	Base change	Protein change	Variant type	Classification
RIN3	NM_024832.5	c.2209C>T	p.P737S	Nonsynonymous Single Nucleotide Variation	Uncertain significance
PLCG2	NM_002661.5	c.32C>T	p.A11V	Nonsynonymous Single Nucleotide Variation	Uncertain significance
KCNJ13	NM_001172416.1	c.80G>A	p.S27N	Nonsynonymous Single Nucleotide Variation	Uncertain significance

© 2025 Priemer DS et al. *JAMA Network Open*.
