# Supplementary material for: Severe Chronic Traumatic Encephalopathy in a US Naval Special Warfare Combatant Crewman
Source: JAMA Netw Open. 2025 Jun 26;8(6):e2517686. doi: 10.1001/jamanetworkopen.2025.17686 (PMC12203286; doi:10.1001/jamanetworkopen.2025.17686)
Supplement: Supplement 2. — Data Sharing Statement [file jamanetwopen-e2517686-s002.pdf]

Data Sharing Statement

Data
Data available: No

Additional Information
Explanation for why data not available: Sharing such data will allow for identification of the subject. We are bound by our IRB protocol to preserve confidentiality of our study subjects.
